# General practitioners uses and perceptions of voluntary electronic feedback on treatment outcomes – a qualitative study

**DOI:** 10.1186/s12875-014-0193-6

**Published:** 2014-11-30

**Authors:** Maria Laura Lippert, Marius Brostrøm Kousgaard, Lars Bjerrum

**Affiliations:** Department of Public Health, The Research Unit for General Practice and Section of General Practice, University of Copenhagen, Øster Farimagsgade 5, P.O. 2099, 1014 Copenhagen, København Denmark

**Keywords:** Denmark, Family practice, Feedback, Quality improvement, Quality indicators, Qualitative research

## Abstract

**Background:**

Currently, there is a strong focus on the diffusion and implementation of indicator-based technologies for assessing and improving the quality of care in general practice. The aim of this study was to explore how and for what purposes indicator-based feedback is used by the general practitioners (GPs) and how they perceive it to contribute to their work.

**Methods:**

Qualitative interviews with nine GPs in two regions in Denmark. The main selection criterion was that the informants had experience with retrieving electronic feedback. The data generation was explorative and open-ended and the analysis took an iterative approach with continuous refinement of themes that emerged from the data.

**Results:**

The study identified two main uses of feedback: i) Administration of a regular disease control schedule for patients with chronic disease and ii) Routine monitoring of outcomes for purposes of resource prioritisation and medication management. Both uses were deemed valuable by the GPs, but also as an additional extra to the clinical core task. All the GPs experienced the feedback to be of limited relevance to the most central and challenging aspects of clinical work understood as the care for individuals. This led to different reactions: Some GPs would use the feedback as a point of departure for broader deliberations about individual patient needs and treatment approaches. For others, the perceived limitations decreased their overall motivation to seek feedback.

**Conclusions:**

The study points to the importance of clarifying limitations as well as possibilities with respect to different aspects of clinical quality when introducing indicator-based technologies to practitioners. The results also emphasize that an indicator-based approach to quality improvement should not stand alone in general practice since some of the most central and challenging aspects of clinical work are not covered by this approach.

## Background

Currently, there is a strong focus on the diffusion and implementation of indicator-based technologies for assessing and improving the quality of care in general practice [1-5]. This is a continuously evolving field as technological and professional developments, together with external demands for transparency and accountability, are pushing health organizations to measure and scrutinize their performance. Many studies have investigated the effects of performance feedback on professional behavior and patient outcomes [6,7]. The mixed results from these studies have spurned increased interest in identifying the key determinants for effective implementation of feedback systems in order to optimize their use at the practice level [6,8]. However, other studies have problematized the increased spread of performance measurement by calling attention to several negative consequences such as ‘measurement fixation’ (professionals focus too much on complying with the indicators at the expense of using contextual judgment to meet the needs of patients), ‘crowding out’ (professionals focus too much on the aspects of care covered by the indicators at the expense of problem areas not covered by the indicators), and overtreatment [9-12]. These consequences are more likely to occur when performance-measurements are linked to financial rewards or sanctions, but the literature also suggests that professional understandings and priorities may be affected by the criteria inscribed in quality assurance technologies with no formal incentives embedded [13,14]. The critique of performance measurement has been especially pronounced in relation to family practice where the assessment of ‘quality’ through a few quantifiable indicators for single diseases are seen to clash with the traditional understanding of proving holistic care around the needs of individuals with complex health problems [15-18]. Due to the controversies surrounding the increased significance of performance measurement there is a continuing need to investigate how outcome feedback is actually used by clinicians in relation to particular aspects of work, and in what sense particular uses of performance feedback are perceived to contribute to improvements [19,20]. Also, since different countries have developed different systems of performance measurement there is a need for detailed studies of these issues in relation to particular systems, so that the ground may be prepared for cross-national comparisons of various approaches to performance improvement. For instance, many studies have focused on pay-for-performance systems in general practice [18,21-23], especially the Quality and Outcomes Framework in the UK. But much less attention has been devoted to investigate how GPs use indicator technologies that are not linked to financial rewards while still relying on performance feedback as a means for improving clinical quality. Therefore, this paper focuses on such a technology, namely the Data Capture Program (DCP) which has been implemented in Danish general practice since 2007. The aim of the study was to explore how and for what purposes feedback from the DCP is used by the GPs and how they perceive it to contribute to improved quality of their work.

### The data capture program and electronic feedback

Clinical performance measurement is a relatively recent phenomenon in Scandinavian primary care. In Denmark, the Data Capture Program (DCP) was developed by a group of GPs with the aim of facilitating research by systematic collection of data on treatment results from general practice clinics. The development of the technology was financed by the University of Southern Denmark and the Danish Ministry of Health. In 2007 the DCP was introduced as a voluntary quality improvement tool to GPs. In return for sending clinical data to a national database, the Danish General Practice Database, DAMD, the GPs get access to electronic indicator-based feedback on outcomes for patients suffering from chronic diseases. The daily operations of the DCP and the DAMD are financed by funds allocated from the general agreement between the regional health authorities and the Organization of General Practitioners.

**Figure 1:**
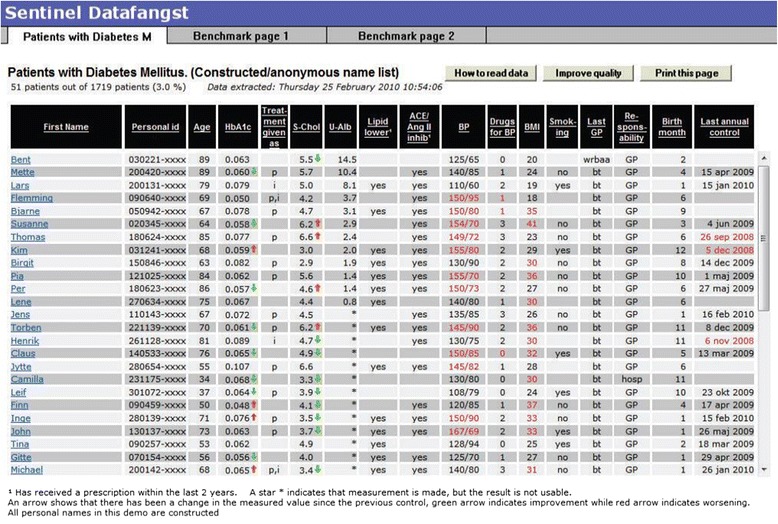
**Example of feedback on outcomes for patients with type 2 diabetes (constructed/anonymous name list).**

The technology is designed to collect data from the clinics without introducing time-consuming registration activity at the practice level. Quantitative information on prescribed medication, diagnoses and laboratory results are automatically collected from the electronic patient records by means of a software module. GPs can retrieve indicator-based feedback on the status of patient treatments by using a digital signature and log-in on the website of the DCP. The feedback provides a total overview of the most recently registered laboratory values (e.g. Hba1C, blood pressure) for all patients registered in the clinic’s electronic system with a specific diagnosis. It also gives an overview of the dates of the clinic’s latest contact with each patient (Figure 1). Thus, administrative and clinical information is presented in joint overviews and it is possible to sort this information according to different criteria: best/worst regulated, date of birth, latest check-up visit, etc. The system also has a benchmark section where the GPs can compare their own treatment results (and the development of these over time) with the municipal, regional and national average of their colleagues.

The first disease areas to be included in the technology were type 2 diabetes and chronic obstructive pulmonary disease, COPD. Subsequently, indicators for more disease areas have been developed. In 2011, the collective agreement made it mandatory for all general practices to sign up to the system (1 April 2013 at the latest). In May 2013, approx. 91% (3276) of all general practices had installed the technology. 36% (1167) of these have logged in to seek feedback at least once [24]. In the coming years, the scope of the DCP is expected to increase when other major disease areas are to be included in the technology and when general practice is to be accredited as part of the Danish Quality Model, DDKM. In contrast to performance measurement systems in UK and USA, no financial incentives have so far been tied to the performance indicators in the DCP. There are several reasons for this. First, as mentioned above, the DCP was originally developed by professionals primarily for purposes of research and quality improvement – not quality control. Second, although pay-for-performance suggestions have been brought up in policy discussions over general practice, such suggestions have not gathered sufficient political backing so far and have been met with opposition from the Organisation of General Practitioners.

## Methods

The results reported here derive from nine semi-structured interviews with GPs in eight general practices situated in the Capital Region of Denmark and in Region Zealand. The interviews were carried out from January to March 2011. They formed part of a multi-sited field study among early voluntary users of the DCP focusing on uses, perceptions and implications of the technology. The study was formulated on the basis of theoretical literature on performance measurement technologies [13,14,25], but the inquires were not directed by a specific theoretical framework [26,27]. An explorative, descriptive and open-ended approach to data generation was chosen in order to remain sensitive to the specific context and object of inquiry [26-28].

### Recruitment

Since the objective of the interviews was to generate extensive and rich information on uses and perceptions of the feedback, the main selection criterion was that the informants had experience with retrieving feedback from the DCP [29,30]. Therefore, information on the frequency of feedback-retrieval was obtained from the Danish General Practice Database, DAMD (total number of log-ins for each healthcare provider identification number). Among healthcare provider identification numbers with between 20 and 100 log-ins since sign-up (n = 22) a short survey was conducted. All GPs (n = 55) in those 22 practices were contacted in writing and asked to answer questions on their motivation for signing up for the DCP, their satisfaction with the technology and their willingness to participate in the present study. Answers were obtained from 32 GPs distributed on 19 practices. The short survey indicated a high level of general satisfaction with the technology. A wish for quality improvement of own practice appeared as the common motivation for adopting the technology. All the GPs responding to the survey were willing to be contacted again. From this population of early voluntary users a purposive sample of nine informants was selected to represent a range of perceptions of the technology, including of its usefulness as a quality improvement tool. Variation on organisational and demographic characteristics was also ensured. A distribution of these characteristics is presented in Table 1. To support this final selection, the information obtained in the survey was further explored through telephone contact and online information.

**Table 1:** **Sample distribution (9 GPs from 8 clinics)**

**Category**	**Variables**	**N**
Gender	Male	6
	Female	3
Age	40-49	2
	50-60	6
	> 60	1
Practice type	Single-handed	3
	Group	6
Practice location	Urban	6
	Rural	3

### Interviews

The interviews lasted 60–90 minutes. All interviews were conducted in the surgeries. In order to give the informants time for reflection, the interviews took place, as far as possible, after working hours and in another room than the consultation room [29]. The interview guide was drafted by the first author, and subsequently discussed and developed within the multi-disciplinary research team (MLL is a sociologist, LB is a GP and Professor of Family Medicine and MBK is a political scientist). The interview guide was constructed on the basis of research literature on practice level responses to and implications of quality measurement technologies [25,31-34], and on written empirical material on the DCP [35-37]. The themes explored in the interviews were also informed by participant observations of formal meetings where the technology had been on the agenda (e.g. Advisory Committee for Patient Episodes of Care and Indicators), and by expert interviews with representatives from the Danish General Practice Database, DAMD. The guide was modified progressively allowing insights from early interviews to inform topics discussed in subsequent interviews. Interview questions covered three main areas: the informants were asked how, when and for what purposes they used feedback in their daily work. Furthermore, they were asked how they perceived their uses of feedback to be related to different aspects of their work, whether, and if so, in what ways their uses of feedback had contributed to increasing the quality of different clinical tasks. Since it had primarily been possible for the GPs to gain experience with feedback concerning type 2 diabetes and COPD, the interviews focused on the use of feedback concerning these disease areas. All interviews were audiotaped and fully transcribed.

### Analysis

An iterative approach to analysis of the interview-material was taken, with constant refinement of themes that emerged from the data. After all the interviews had been conducted the transcripts were read several times to get a sense of the material in its entirety and emerging themes were discussed within the research team. The first systematic mapping and formulation of analytical categories [38] was performed by the first author. The categorization of the material started with the identification of uses of feedback for particular purposes and reflected the questions asked during the interviews. Themes and sub-themes regarding the GPs’ reflections on those uses and their overall perceptions of the technology were extracted from the material during a process of deeper level thematic coding. Themes and patterns were identified using open approaches to coding without preconceived categories [39], but the process involved comparisons with findings from studies of responses to similar technologies in primary care settings in the UK [25,31-34] and with literature on quality and task perceptions in general practice [16,40,41]. Based on the initial coding performed by the first author themes and sub-themes were added or amended in a shared process between the researchers. The coding of the interviews was refined after a subsequent period of study that included follow-up questions to the informants in order to validate the findings. This period of study ended with the assessment that data saturation [42] regarding particular uses of feedback had been achieved. Each GP expressed their personal reflections on their uses of the technology. An overall thematic pattern linking those individual reflections to a shared perception of the professional task was however emerging so strongly from the material, that is was decided not to include more informants in the study.

The project was declared to the Danish Data Protection Agency. No ethical approval was needed for the study according to The Danish Ethical Committees. Participants were GPs only, and the focus of the study was on clinical routines and perceptions, not biomedical research on humans. The identities of the surgeries and the GPs have been concealed in order to protect their anonymity.

## Results

The study identified two main uses of electronic feedback: i) Administration of a regular disease control schedule for patients with chronic disease and ii) Monitoring of patients for purposes of resource prioritisation and medication management. Those two uses of feedback were described as routinely and as primarily based on the feedback’s standard-indications. Some GPs also used the feedback as a point of departure for broader considerations about individual patient needs and specific treatment approaches and linked interpretations of feedback data to other sources of knowledge. In the following, we first elaborate on the different types of use and then on the variations in the perceptions of feedback related to these differences in use.

### Administration of a regular disease control schedule for patients with chronic disease

Most informants described how they used the feedback to support the administration of a regular disease control schedule for patients with chronic diseases, as recommended by the Danish College of General Practitioners (DSAM) since 1991 [43]. For type 2 diabetes, this control schedule typically involves three annual check-ups with a nurse and an annual check-up with the GP. When the feedback was described by the informants in connection with such purposes of administration, it was commonly referred to as a *checklist* or as a quality *assurance* tool.

Some informants had already implemented this control schedule for their diabetes and (to a lesser extent) COPD patients prior to signing up for data capture. These GPs regarded the feedback’s overview of birth dates and latest check-up visits as useful for identifying patients who had not been scheduled to a fixed control or who had not showed up for appointments. Apart from this, these GPs did not experience much change, but often described the control of their chronic patients as more regular or more systematic:

*We haven’t changed a bit! We have just become more systematic. We now have the kind of overview that facilitates that we check whether somebody has let us down and not shown up for controls. I think it is a brilliant tool for that…. I just print this and then the secretary makes sure the patient receives an appointment.* [GP # 2]

In practices that had not already incorporated a regular control schedule before the implementation of data capture, the feedback had also worked as a tool for introducing the recommended schedule of controls. In these practices, the informants described how the access to the feedbacks’ population overviews and the benchmark data on average prevalence of diagnosis had made them more aware of diagnostic coding and had led them to increase diagnostic coding of patients with type 2 diabetes. These GPs experienced that they now managed to get *more patients through the system*. Some of them had adopted the technology specifically because of a wish to implement *a framework for regular controls of chronic patients*:

*This was what we had been missing…. We really had hesitated a bit and been in doubt as to how we should do things. Suddenly there was a box, which could be implemented directly in practice. By Jove, this was clever!* [GP #1]

It was a general assessment among the informants that the access to feedback had provided them with a better overview of their chronic patient populations. Depending on practice organisation, routines and individual preferences, the use of the feedback as administrative information for managing controls of chronic patients was either taken care of by the GPs themselves or partly delegated to practice staff.

### Monitoring patients with chronic disease

For most informants, the use of feedback to administrate a systematic control schedule was combined with more or less routine monitoring of treatment conditions for patients with chronic diseases based on the feedback’s overview of patient outcomes. When the feedback was described in relation to this monitoring, it was often referred to as a *“checklist”* or a *“script”.*

The main purpose of outcome monitoring was to distinguish between medically well-regulated and less well-regulated patients. That differentiation was associated with objectives of resource prioritisations and of medication management:

#### Monitoring for resource prioritisations

The GPs in the study received a fixed annual fee – a so-called episode-of-care payment or bundled payment – for each patient with type 2 diabetes. This fee differs from the normal remuneration for individual contacts and is supposed to cover the various amounts of work in relation to each patient. Several informants explained that they could gain financially by using the feedback to distinguish between medically well-regulated and less well-regulated diabetes patients and by using this information to adjust the number of additional check-ups for each patient:

*Probably, we have become more systematic in the sense that we now take more into regard, at the annual check-up, whether the fixed number of check-ups is actually a reasonable number* […]. *It makes some sense financially, when you receive a fee for the whole year and you may only need to see them once or twice a year – rather than being forced to see them four times* [GP #3]

Whilst the administrative use of feedback described in the first section supported the *adaption* to a fixed control schedule, the use of feedback to monitor outcomes could often support resource related decisions to *deviate* from the standard schedule. This approach to prioritisation – need-stratification based on outcomes – was described by the informants as being novel to them.

#### Monitoring for medication management

Most GPs found that their use of feedback for monitoring outcomes helped to make them aware of patients who were poorly medically regulated. This could result in additional check-ups or in decisions to change or increase the medication for these patients. According to one informant, the use of feedback had resulted in an overall enhanced structure of the medical treatment, which he described as separate from deliberations on treatment strategies:

*Having access to the actual data has convinced us that some of our patients are too poorly regulated: now they are in for maximum medical treatment. It’s not that I have become better at choosing other drugs or treatment strategies or the like, but the system has a script-like quality, which is really okay.* [GP # 1]

It was a shared feature of the above described forms of monitoring that they were predominantly based on the feedback’s indications of satisfactory or unsatisfactory outcomes of treatment. A few GPs related that they – for reasons of mere curiosity – sometimes visited the feedback’s benchmark section to compare results of treatment with the averages of colleagues. One GP related that looking up the benchmarked data aroused his competitive inclinations, but that it did not affect his clinical priorities since he was always “*in front*”.

### Broader deliberations about individual patient needs and treatment approaches

Some of the GPs described how looking up feedback could lead to broader and more thorough deliberations about the management of individual patient cases. In that context, the feedback was not described as a checklist or a script, but for instance as a “*dialogue partner*” or as an occasion for “*professional reflection*”*.* As an example, such deliberation might concern the appropriateness of medical regulation as a treatment strategy in specific patient cases:

*You hold your horses, you know, and take a look at the patient. What is appropriate for you etc.… I am simply not willing to make the attempt to beat some patient’s HbA1c down to a very low level if I’m convinced it is not the proper approach in that specific case.* [GP # 4]

*I might easily just print this chart* [feedback] *and then take a look at the patients’ data to check why a patient is not getting cholesterol-lowering medication. Is it because we haven’t been thinking straight or is there a good reason for it?* [GP # 2]

According to the informants, such reflections always included more knowledge about the patients than was provided by the feedback. This could be knowledge about a patient’s (current) life situation, his or her individual preferences and concerns, or the presence of co-morbidity. Such assessment processes were not described as different in content from those, which the same GPs would have carried out without access to feedback. However, several informants reported that the feedback’s overview of chronic patient populations had become a new occasion for this kind of reflections. Several GPs told that the feedback had helped to make them more aware of treatment-related problems in patients who had special needs and might not have visited their doctor on their own account.

### Variations in the use and perceptions of feedback

The occasional use of feedback as a basis for broader reflections on individual patient cases was particularly described by GPs who took a special interest in a specific chronic disease area (or in chronic disease management in general) and/or by GPs from practices, where a mutual decision had been made to give priority to that kind of use. In one partnership practice, the decision to implement the DCP included a decision to give priority to “*professional reflection*” by allocating a monthly number of hours to each doctor. Another partnership practice had scheduled a couple of annual meetings dedicated to discussions of clinical issues on the basis of the feedback.

Approximately half of the GPs reported that their use of feedback did not extend beyond administration and routinely monitoring of outcomes. Those doctors often described their use of feedback as being separate from “*strictly professional*” purposes:

*Well, I never use it for anything professionally. We use it for some basic, common things. Such as when they* [the patients] *need to come, and how it’s all going, and who takes care of what […] It’s great to have a structure to it, and it does make some financial sense, but we haven’t really used the clinical information.* [GP # 5]

*It’s difficult for us, the thing about using it for strictly professional purposes. When you ask others how much they use it, people tend to look the other way. You hardly ever get round to it…* [GP # 6]

What was commonly referred to as a “*strictly professional*” use of the feedback turned out to cover overall clinical deliberations on prioritisations and specific treatment approaches as well as assessments of individual patients’ needs. The reported reasons for not giving priority to such uses included lack of time and cooperation problems in the clinics. However, the reasons also included a shared perception of the feedback as insufficient for assessing individual cases and of a disparity between what appeared in the feedback as satisfactory treatment and what – based on the GPs own professional assessment – could be considered as satisfactory treatment. Such disparities were always explained with reference to the GPs’ contextual knowledge about patients:

*Here* [in the feedback] *you can only see that her diabetes is well-treated, but she is not well treated for her lifestyle or her competing factors or anything else […] She just hasn’t got a good life, but you simply can’t see that here!* [GP # 5]

Independently of how they used the feedback, there was a shared perception among the GPs that the information presented in the feedback did not concern the most important and challenging aspects of the clinical work understood as the care of and personal contact with individual patients:

*There are so many other important aspects of my work, which cannot be measured in the same way as long-term blood glucose levels. The other sort of quality grows out of the ordinary and demanding daily work. The really, really difficult bit is the lifestyle thing, but that is not really evident from this [the feedback]. That is the challenging part…* [GP # 2]

The informants displayed different reactions to the perceived limited relevance and incomplete presentation of patients’ health condition. Some GPs simply operated with a dual definition of quality: “*Quality as defined by the feedback data*” on the one hand and “*the reality with the patients*” on the other. Those GPs described their efforts to give priority to both kinds of quality and to maintain a balance:

*We have experienced becoming prone to focus too strongly on data. That way it easily becomes an impediment* […]. *We have to remember the other things…. Quality is just as much about talking properly with the patients. Data capture comes with the risk that it may make you concentrate on measurable aspects and forget about everything else.* [GP # 7]

For others, the experience of a disparity between good clinical quality according to the feedback and good clinical quality according to their own professional judgment had led to disappointment and frustration with the technology, and had decreased their overall motivation to look at feedback. As a consequence, one practice had abandoned the use of feedback for anything else than administration delegated to practice staff.

## Discussion

### Principal findings

This study identified various uses of feedback on outcomes. The two most common uses (i.e. to implement/manage a regular disease control schedule for chronic patients and to monitor outcomes of medical treatment) were perceived to be beneficial by most of the GPs. The feedback was deemed valuable by most informants because it provided them with standards and checklists for systematizing overall clinical priorities and for gaining an overview, which supported a proactive and resource-related approach to chronic care and made financial sense in connection with a bundled payment. Those uses of feedback were, however, described by all the informants as an additional extra to the professional core task, understood as the care for individuals. All the GPs perceived the feedback on outcome as insufficient for evaluating the actual quality of care and the need for treatment in individual cases. Their different responses to this shared perception varied from *i)* attempts to deal with and prioritise attention to feedback data and the care for individual patients as distinct concerns, existing alongside each other to *ii)* attempts to integrate the feedback in broader assessments of individual cases and *iii)* partly ignoring the feedback.

### Limitations of the study

Two potential limitations of this study can be mentioned: First, the study was carried out among a small group of pioneers and early adopters [44,45] who had voluntarily installed the technology. Most of these GPs had adopted the DCP specifically because of a wish to systematize their chronic care management and all of them described high motivations for this kind of quality improvement of their own practice. Also, the use of feedback for administrative purposes was often supported by already existing organizational routines in these clinics. Such favourable pre-conditions for implementation in terms of motivations and organizational routines cannot be expected to be found in all clinics who adopt the technology primarily to comply with public regulatory requirements [46]. On the other hand, the informants in this study do form a basis for generalisation in the sense that the reservations articulated by these highly motivated early adopters with a pronounced interest in this kind of quality improvement are also likely to be found among a broader population of GPs with various dispositions towards an indicator-based approach to quality improvement [45].

Second, employing interviews to investigate an organisational practice (the use of feedback) have certain limitations related to recalling problems [47] and the interplays between researcher and informants – impression management [48]. Therefore, direct observation could be proposed as a supplementary method to strengthen validity in this regard. However, since the GPs retrieval and use of feedback is sporadic and dispersed in time (before, during, or after opening hours) and space (the clinic or at home) observation would be very difficult and time-consuming.

### Comparison with other studies

One of the most frequently mentioned factors influencing the effectiveness of feedback is motivation for adoption among the recipients [49]. The present study adds to this by pointing to the significance of recipients’ motivations being based on realistic expectations regarding the scope of indicator-based measurements: All the GPs included in this study were highly motivated, but those who expected the feedback to provide a full picture of individual patients’ health condition described disappointment and decreased motivations to seek feedback. Those GPs who expected the use of feedback to require time, reflection and inclusion of other criteria than those presented by the technology were generally more inclined to seek feedback and to perceive their use of this information as integral to the professional task.

In line with other research [16-18,31-34,40], the present study found tensions between the outcome indicators for single diseases and the GPs’ more holistic understanding of the professional task. Critical studies on implications of performance measurement technologies in primary care settings have reported on negative effects in the shape of displacement of contextual and reflective professional approaches [17,32-34]. The present study did not identify these kinds of implications: The GPs’ uses of feedback in assessments of individual cases were informed by reflections regarding the need to contextualize and interpret standard-indications. The feedback was, however, most commonly used to support overall administration of chronic care since administrative tasks were perceived by all the informants as the most obvious aspect of their work to benefit from attention to routine data and a checklist approach. This is in line with studies from the UK suggesting that ambivalence towards standardized data may lead GPs to restrict their use of feedback to purposes that are meaningful to them while side-lining other official objectives [15,31]. The present study adds to this knowledge by showing how such perceptions of meaningfulness may differ in relation to different aspects of professional work: overall administrative and managerial tasks and the assessment of individual cases respectively.

## Conclusion

Indicator-based feedback is being used to support overall administrative and managerial tasks in the clinic, which are increasingly encouraged in primary health policies. The present study points to the potential usefulness of electronic feedback to support prioritization of such aspects. However, the study points to the importance of clarifying the limitations and the requirements of use (including the need for active and contextual interpretation of routine data) when introducing this kind of quality improvement technology to GPs [14,10]. The fact that none of these highly motivated GPs regarded feedback on outcomes as contributing to improved quality of the most important and challenging aspects of their work emphasize the importance of not letting an indicator-approach to quality improvement stand alone in general practice. This study and current moves to expand the scope of indicator-technologies to include more disease areas and more aspects of clinical practice suggests several avenues for further research: First, it is important to explore how GPs respond to performance feedback when it comes to disease areas that are more difficult to quantify via biomedical indicators (such as stress, anxiety and depression) [49,50]. Secondly, it is relevant to study how attention to feedback on performance affect professional prioritisations and the interaction between doctors and patients during consultations [17,32-34].

## Abbreviations

COPD: Chronic obstructive pulmonary disease
DCP: Data capture program
GP: General practitioners
DAMD: Danish general practice database
DAK-E: Danish general practice quality development unit
DDKM: Danish quality model
